# The dual role of autophagy in suppressing and promoting hepatocellular carcinoma

**DOI:** 10.3389/fcell.2024.1472574

**Published:** 2024-10-11

**Authors:** Wasnaa H. Mohammed, Ghassan M. Sulaiman, Mosleh M. Abomughaid, Daniel J. Klionsky, Mohammed H. Abu-Alghayth

**Affiliations:** ^1^ Department of Biotechnology, College of Applied Sciences, University of Technology, Baghdad, Iraq; ^2^ Department of Medical Laboratory Sciences, College of Applied Medical Sciences, University of Bisha, Bisha, Saudi Arabia; ^3^ Life Sciences Institute, University of Michigan, Ann Arbor, MI, United States

**Keywords:** autophagy, autophagy dual role, cancer, drug resistance, hepatocecllular carcimoa

## Abstract

The 5-year survival rate for hepatocellular carcinoma (HCC), a deadly form of liver cancer, is quite low. Although drug therapy is successful, patients with advanced liver cancer frequently develop resistance because of the significant phenotypic and genetic heterogeneity of these cells. The overexpression of drug efflux transporters, downstream adaptive responses, malfunctioning DNA damage repair, epigenetic modification, the tumor microenvironment, and the extracellular matrix can all be linked to drug resistance. The evolutionary process of autophagy, which is in charge of intracellular breakdown, is intimately linked to medication resistance in HCC. Autophagy is involved in both the promotion and suppression of cancer by influencing treatment resistance, metastasis, carcinogenesis, and the viability of stem cells. Certain autophagy regulators are employed in anticancer treatment; however, because of the dual functions of autophagy, their use is restricted, and therapeutic failure is increased. By focusing on autophagy, it is possible to reduce HCC expansion and metastasis, and enhance tumor cell reactivity to treatment. Macroautophagy, the best-characterized type of autophagy, involves the formation of a sequestering compartment termed a phagophore, which surrounds and encloses aberrant or superfluous components. The phagophore matures into a double-membrane autophagosome that delivers the cargo to the lysosome; lysosomes and autophagosomes fuse to degrade and recycle the cargo. Macroautophagy plays dual functions in both promoting and suppressing cancer in a variety of cancer types.

## Introduction

The predominant liver cancer subtype, which ranks second in terms of approximate mortality after pancreatic cancer, is hepatocellular carcinoma (HCC) (Siegel et al., 2020). The survival rate after 5 years for HCC is 18%. Treatments for HCC include surgical resection, liver transplantation, chemotherapy, radiofrequency ablation, targeted therapy, transarterial chemoembolization, and immunotherapy (Villanueva, 2019). Nevertheless, the majority of individuals with advanced or intermediate stages of HCC do not effectively react to anticancer medications, and only a small percentage of individuals are susceptible to these treatments. In HCC patients where medication resistance has already developed, the total survival rate is decreased (Llovet et al., 2008). To save HCC patients, it is crucial to understand HCC’s mechanism of drug resistance, pinpoint the targets of drug resistance, and optimize the therapy regimen.

HCC is a naturally drug-resistant tumor. Drug resistance is caused by the transmembrane ATP-binding cassette (ABC) transporter protein, which prevents anticancer medications from penetrating cells. Drugs are delivered to cancer cells by inhibition of the solute carrier (SLC) family, which also leads to multidrug resistance (MDR) development in HCC cells (Lei et al., 2024), chemotherapeutic medications typically have little effect on HCC patients. During treatment, the associated tumors are likely to develop MDR, which lowers survival and worsens the prognosis (Wang et al., 2014; Koltai, 2022). The restricted accumulation of anticancer medicines within tumors is one of the factors behind resistance to these medications. In this sense, pharmaceuticals are transported across the cellular membrane via membrane transporters, which are primarily responsible for the influx (mostly by members of the SLC transporter family) and efflux (primarily by members of the ABC transporter family). Thus, transporters control the concentrations of drug at the target site in both healthy tissues and cancer cells, which has an impact on therapeutic results. One of the main challenges in anticancer drug delivery and a major factor in the failure of cancer medication therapy is transporter-mediated drug resistance. Because SLC transporters are essential for the drug’s intracellular absorption drug therapy may be rendered ineffective if cancer cells exhibit downregulated expression and function of SLC transporters, which restricts the absorption of medications into the tumor cells. The phenomenon known as multidrug resistance occurs when a high percentage of patients become non-responsive to numerous functionally and structurally varied anticancer medications as a result of developing drug resistance to chemotherapy and molecularly targeted therapies (Puris et al., 2023). Exogenous and intrinsic factors are the two main causes of medication failure in cancer patients. For example, the result of genetic changes that were already present in tumor cells prior to the initiation of therapy is considered intrinsic resistance, sometimes referred to as primary resistance. Included in this are treatment resistance linked to cancer stem cells/CSCs (Chan et al., 2015), as well as elevated expression of drug efflux transporters (Alisi et al., 2013), which bind and eliminate chemotherapeutic agents. Exogenous resistance, sometimes referred to as developed resistance, occurs when cancer cells that are originally responsive to drugs later become resistant to them following a course of chemotherapy (Chan et al., 2015).

Autophagy is a system that has been maintained over evolution. It plays a role in the turnover of organelles and proteins, as well as in the regulation of metabolism and cell quality control (Kumariya et al., 2021; Raudenska et al., 2021). Lysosomes are necessary for the process of autophagy, which has as its primary objectives maintaining cellular quality control and the production of energy and nutrition through the breakdown and release of cytoplasmic substituents. To maintain cellular health and balance, the autophagy-lysosomal system—a combination of autophagy and the lysosomal system—eradicates waste products from cells, including protein aggregates, damaged organelles, and invasive microbes. Consequently, dysfunction of the autophagy-lysosomal system contributes to several pathophysiological states, including neurological disorders, cancer, inflammatory and immunological diseases, and metabolic abnormalities (Kumar et al., 2021).

Additionally, autophagy is regulated to protect cells from various types of stress. Including malnutrition, hypoxia, DNA damage, chemotherapeutic exposure, and meeting cells’ metabolic demands to keep organelles and cellular signaling pathways intact (Glick et al., 2010; Parzych and Klionsky, 2014). The human body uses autophagy frequently in response to different stimuli (Huang F. et al., 2018). The primary routes of autophagy can be broadly categorized into five stages: initiation, expansion, closure, maturation, and degradation; the final step involves the release of breakdown products back into the cytoplasm (see below under Autophagy basic mechanism). Cellular homeostasis is maintained through autophagy by the elimination of improperly folded and aggregated proteins, and damaged organelles (He and Klionsky, 2009). Autophagy also plays a crucial role in the immune response, for example, by regulating the activation of inflammasomes by primary macrophages generated from bone marrow (Jabir et al., 2014; Jabir et al., 2021).

## Hepatocellular carcinoma

In terms of mortality, hepatocellular carcinoma, a type of primary liver cancer, is ranked second and according to the frequency of occurrence is the seventh globally (Bray et al., 2018; Katherine et al., 2021). HCC is the most prevalent kind of liver cancer, comprising up to 75% of cases. The top rates of liver cancer occurrence are discovered in Asia and Africa (Zhang et al., 2023). Up to a half million new cases of HCC are identified each year, contributing to a high mortality rate (Tsochatzis et al., 2014; Petrick et al., 2020). Five percent of patients with HCC survive longer than 5 years after the initial diagnosis, which is a relatively poor survival rate. This poor prognosis is connected to the fact that only 15% of these patients are eligible for or capable of undergoing surgery and liver transplantation due to the late identification of HCC. Thirty-five percent or more of HCC patients receive the greatest care at the time of diagnosis, and fifty percent have non-surgical therapy (El-Serag, 2011; Yun et al., 2020). In hepatotoxicity, usually associated with different liver injuries (Hashemi et al., 2023), the oxidative process, which is thought to be the primary factor causing a shift toward inflammation, fibrosis, and hepatocellular damage, is triggered by free radical damage and many other factors, which lead to HCC (Mohamed et al., 2020a; Mohamed et al., 2020b). Race, gender and age are factors that affect the chance of getting HCC. Men are two to four times more likely than women to develop HCC. Up to 75 years of age, HCC exhibits a positive relationship with age (Petrick et al., 2020). Hepatitis virus infection, alcohol consumption, nonalcoholic fatty liver disease and cirrhosis are among the risk factors for this disease (Mohamed et al., 2022).

HCC progression is influenced by various molecular pathways and processes (Deldar Abad Paskeh et al., 2021; Yu et al., 2021). For example, FIS1 (fission, mitochondrial 1) provides one example of how the cellular machinery for mitochondrial fission intersects with HCC. This protein was thought to have a critical role in mitochondrial fission; however, mammalian cells lacking FIS1 do not exhibit an obvious defect in this process (Yamano et al., 2014; Wong S. W. et al., 2018). Nonetheless, FIS1 phosphorylation via MET (MET proto-oncogene, receptor tyrosine kinase) at Y38 facilitates mitochondrial fission by recruiting the mitochondrial fission GTPase DNM1L/Drp1 (dynamin 1 like) to mitochondria (Yu et al., 2021; Zhang et al., 2021); however, it has not been established that FIS1, an outer mitochondrial membrane protein, is a DNM1L receptor (Otera et al., 2010). HCC cells can metastasize both *in vitro* and *in vivo* by the development of lamellipodia or invadopodia, which is facilitated by fragmented mitochondria that drive actin filament remodeling. By activating MET kinase, which directly affects mitochondrial fission as described above, HGF (hepatocyte growth factor) plays a crucial role in the migration and invasion of cancer cells in the HCC microenvironment. Based on this, MET-targeted inhibitors have been used in HCC clinical trials, and FIS1 has been identified as a novel, significant downstream target that is regulated by MET kinase (Deldar Abad Paskeh et al., 2021).

The inflammatory state of the HCC microenvironment makes HCC immunotherapy modalities such as immune checkpoint inhibitors, anti-CTLA4 (cytotoxic T-lymphocyte associated protein 4), and anti-PDCD1/PD-1 (programmed cell death 1) potentially helpful. These alterations may result in changes in the number of regulatory T cells, the induction of dendritic cells, and the release of immune-modulating factors (Shokoohian et al., 2021; Minaei et al., 2023). However, the effectiveness of chemotherapy and radiation therapy in extending the life expectancy of HCC patients is hindered by the formation of several resistance mechanisms during treatment (Niu et al., 2020; Xia et al., 2021; Seydi et al., 2023).

The plasma membrane protein CD44 (CD44 molecule (IN blood group)), which functions as a cell-cell and cell-stromal adhesion protein and is frequently overexpressed in tumor cells, is a marker of poor survival in the majority of solid tumors (Wan et al., 2014). CD44 is linked to tumor malignancy and denotes a poor prognosis for a number of cancers, including liver cancer (Dhar et al., 2018). One of the main factors contributing to a worse prognosis is extrahepatic metastasis (EHM), which can happen when a patient has advanced HCC at the time of presentation or at the time of recurrence (Becker et al., 2014; Yoon et al., 2020). One major obstacle to increasing HCC patients’ overall survival (OS) is the existence of EHM. For individuals with HCC, CD44 is a strong predictor of both EHM and OS. In HCC cells and patient specimens with a high risk for malignancy, CD44 is abundantly expressed. Patients with HCC who overexpress CD44 have a lower OS and a greater cumulative recurrence rate compared to those with low levels of CD44 expression. Experiments conducted *in vitro* and *in vivo* demonstrate that CD44 downregulation inhibits the formation, migration, invasion, growth, and metastasis of HCC cells. Additionally, the pro-metastasis effect of CD44 is mediated through the MAPK/ERK (mitogen-activated protein kinase)-AKT/protein kinase B (AKT serine/threonine kinase)-CXCR4 (C-X-C chemokine receptor 4) axis. The aggressive clinical characteristics of HCCs are compatible with CD44’s documented ability to stimulate CXCR4 expression and increase the tendency of tumors to infiltrate and metastasize to distant organs (Xie et al., 2022). Finally, in HCC, the overexpression of CD44 facilitates the growth and migration of HCC cells through the action of oncogenic YAP1 (Yes1 associated transcriptional regulator), a crucial downstream regulator in the Hippo pathway. These results imply that CD44-YAP1 is most likely a significant axis in the pathophysiology of HCC, offering insights into the etiology of HCC and possible targets for HCC treatments (Yu et al., 2021). Obesity, PIK3CG/PI3Kγ (phosphatidylinositol-4,5-bisphosphate 3-kinase catalytic subunit gamma) ablation, ZRANB1 (zinc finger RANBP2-type containing 1), and *ADORA2A-AS1* (ADORA2A antisense RNA 1) all contribute to HCC development. There are genetic factors that contribute to the development of HCC. PIK3CG/PI3Kγ, a type of phosphatidylinositol 3-kinase, is often overexpressed in HCC and promotes tumorigenesis. ZRANB1, a zinc finger protein, is downregulated in several types of cancer, including HCC. *ADORA2A-AS1*, an antisense RNA, is an oncogenic factor that regulates gene expression and is associated with tumorigenesis. Abnormalities in these genes may play a crucial role in the development of HCC. To fully understand their roles, further research is needed (Becattini et al., 2021; Zhang et al., 2021).

## Autophagy basic mechanism

**FIGURE 1:**
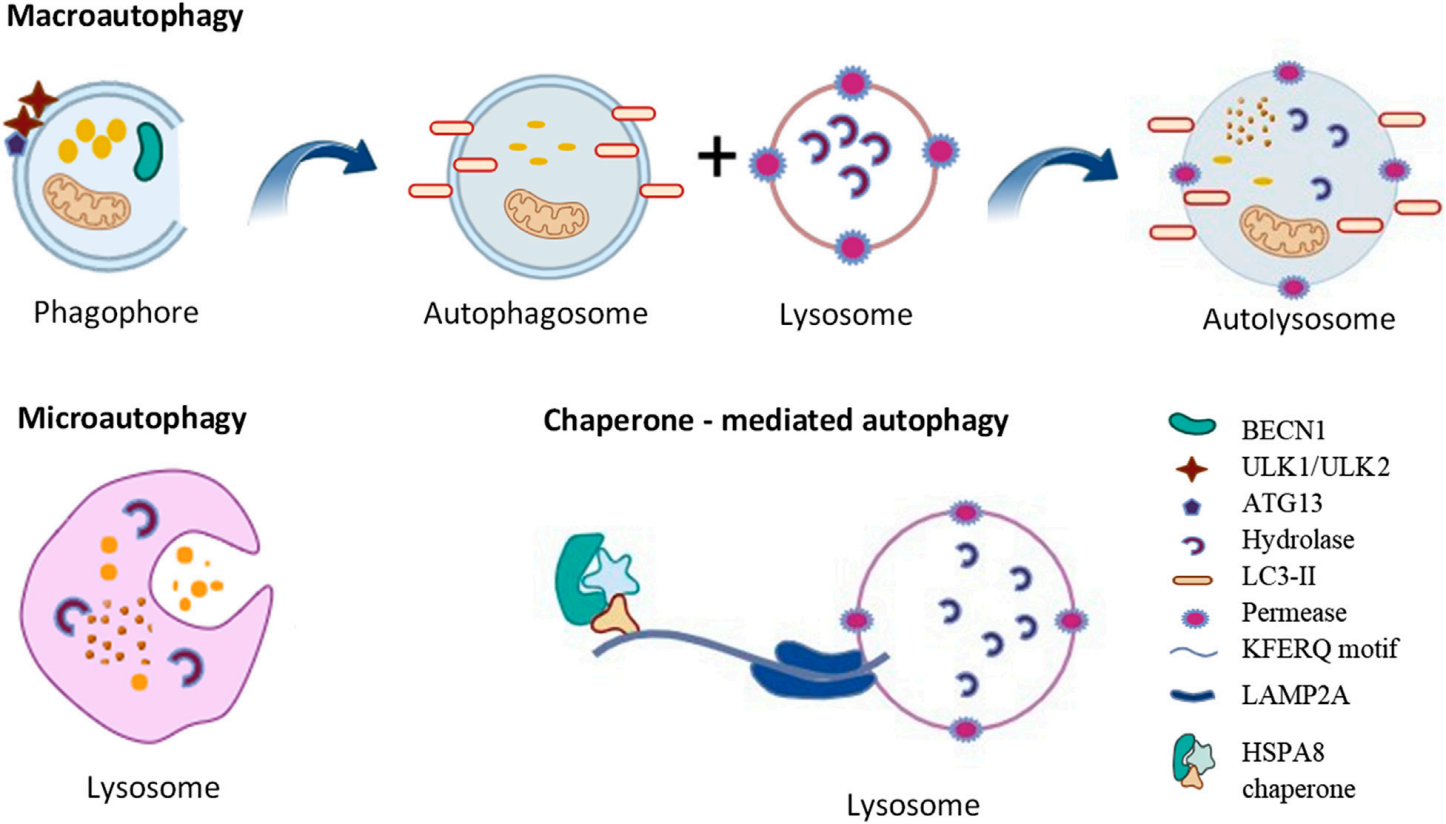
Types of autophagy. See the text for details.

Autophagy is a self-degradation and internal recycling mechanism that is highly evolutionarily conserved, carrying out metabolic requirements and upholding homeostasis (Li Q. et al., 2021). As a homeostatic mechanism, autophagy facilitates the proteolytic breakdown of large cytosolic cellular constituents and aggregates in lysosomes, particularly those that are not susceptible to ubiquitin-proteasome pathway-mediated degradation (Pu et al., 2021). Different kinds of cellular stressors, such as cellular injury, the synthesis of defective proteins, and hunger, and the presence of excess or damaged organelles, pathogens and some microorganisms can trigger the autophagic process (White et al., 2015). Chaperone-mediated autophagy (CMA), microautophagy, and macroautophagy are the three primary forms of autophagy (Figure 1). Microautophagy is a particular kind of direct autophagy in which cargo is captured and cytosolic components are invaginated directly at the lysosomal membrane (Jabir et al., 2018). Lysosomal membrane receptors, that include LAMP2A (lysosomal associated membrane protein 2A), detect and translocate cargo in the form of individual proteins that are complexed with chaperone proteins (such as HSPA8/HSC70 [heat shock protein family A (Hsp70) member 8]); these substrates are unfolded as they cross the membrane into the lysosomal lumen during CMA, a type of selective autophagy that involves the recognition of a KFERQ amino acid motif within the cargo (Yun and Lee, 2018).

The best characterized of these autophagic processes is macroautophagy (referred to as autophagy hereafter). Once cytoplasmic cargo is isolated and incorporated into phagophores, the latter mature into double-membrane autophagosomes. Phagophore nucleation, which is the first step in autophagy, is brought on by the stimulation of the ULK1 (unc-51 like autophagy activating kinase 1) kinase complex, which includes ATG13 (autophagy related 13), ATG101 and RB1CC1/FIP200 (RB1 inducible coiled-coil 1). Expansion of the phagophore involves additional ATG (autophagy related) proteins including the ubiquitin-like proteins ATG12 and yeast Atg8 homologs (composed of the MAP1LC3/LC3 [microtubule associated protein 1 light chain 3] and GABARAP [GABA type A receptor-associated protein] subfamilies) and associated components that are required for their conjugation. The ATG2 (autophagy related 2)-ATG9 (autophagy related 9) complex also plays a critical role in delivering lipids for membrane expansion. Upon completion of sequestration, autophagosomes form. The autophagosomes fuse with lysosomes to create autolysosomes, which perform the breakdown and recycling of the cargo (Yorimitsu and Klionsky, 2005). It has been proposed that ATG2, a rod-shaped protein, is necessary for phagophore expansion because it tethers phosphatidylinositol-3-phosphate-enriched phagophores to the endoplasmic reticulum (ER) (Maeda et al., 2019). Because ATG2 mediates lipid transfer and re-equilibrates membranes in conjunction with ATG9 to facilitate autophagosome formation, it plays a crucial function in autophagy (Wang et al., 2024).

Small vesicles containing the membrane protein ATG9 seed the creation of the phagophore (Maeda et al., 2020; Guardia et al., 2020). ATG9A scramblase moves phospholipids delivered by ATG2 across both sides of the phagophore membrane. Furthermore, it appears from molecular dynamics simulation that lipids can flip across the bilayers because a central pore in Atg9 widens laterally to make room for lipid headgroups. Based on cryo-EM, fungal Atg9 forms a homotrimer containing two linked pores that create a pathway between the two membrane leaflets: one pore at the protomer opens laterally to the cytoplasmic leaflet, while the second pore at the trimer center travels vertically through the membrane (Matoba et al., 2020). At the growing margin of the phagophore, where Atg2 obtains phospholipids from the endoplasmic reticulum, Atg2 and Atg9 colocalize. Lipid scrambling by ATG9A is necessary for membrane growth, as evidenced by the fact that mutations in the pore decrease scrambling activity and result in noticeably smaller autophagosomes.

Although autophagy is primarily a cytoprotective mechanism, cell death may eventually ensue from an overabundance of autophagy and cellular self-consumption brought on by cellular damage—autophagy is considered as one type of programmed cell death (Oku and Sakai, 2018).

A number of autophagy-related proteins, including BECN1 (beclin 1), MAP1LC3/LC3 (microtubule associated protein 1 light chain 3), and ULK1/ULK2 (Table 1) play a critical role in this process. The development of the autophagosome requires ATG7, the ATG12–ATG5 conjugate, Atg8-family proteins such as LC3, and SQSTM1/p62 (sequestosome 1). RABs, SNAREs, and tethers work together to cause lysosomes to fuse with autophagosomes so they can break down cargo and return nutrients to the cytoplasm (Huang F. et al., 2018). Autophagy breaks down the multifunctional protein SQSTM1, which is important in cell development, survival, and death. Tumor development has been linked to *SQSTM1* gene amplification as well as abnormal SQSTM1 accumulation and phosphorylation. By activating the transcription factor NFE2L2/Nrf2 (NFE2 like bZIP transcription factor 2), phosphorylation of SQSTM1 at Ser349 routes glutamine into glutathione production and glucose toward the glucuronate pathway. These alterations confer proliferative potency and resistance to anti-cancer medications on HCC cells. An inhibitor of phosphorylated SQSTM1-dependent NFE2L2 activation blocks HCC growth and resistance to anticancer drugs. In individuals with HCV-positive HCC, an NFE2L2 inhibitor may be able to reduce cancer cell resistance to anticancer medications (Saito et al., 2016).

**TABLE 1:** Key molecules related to autophagy.

Functional units	Role or function	Ref.
• The components of ULK1 kinase complexes		
ATG13	An adaptor that mediates the interaction between RB1CC1 and ULK1 and increases the activity of ULK1 kinase	Li Y. et al. (2021), Alers et al. (2014)
RB1CC1/FIP200	A scaffold protein that interacts with both ATG13 and ULK1 and is necessary for proper localization, stability, and kinase activity of ULK1	Chano et al. (2010), Li et al. (2018), Yi et al. (2023)
ULK1	A protein kinase essential for triggering autophagy in response to different signals. When ATG13 and ULK1 are phosphorylated by MTORC1, bulk autophagy cannot begin. ULK1 activates PtdIns3K via phosphorylating BECN1, a subunit of the complex	Yuan et al. (2019), Lin and Hurley (2016)
• The components of BECN1 core complexes		
BECN1	Autophagy regulation and its role in cancer Important autophagy regulator in a cascade that controls apoptosis, vacuolar protein sorting, endocytic trafficking, and cellular homeostasis	Tran et al. (2021), Ye et al. (2023)
PIK3C3	ARF transcription is inhibited by PIK3C3, which also makes EGFR nuclear transport and binding to the ARF promoter easier Liver CSC expansion is facilitated by PIK3C3 overexpression, but siRNA-induced PIK3C3 knockdown has the reverse effect	Chu et al. (2021), Song et al. (2023)
PIK3R4	A crucial molecule that is involved in many cancers through controlling autophagy. Potentially stops neuronal deterioration and the development of amyotrophic lateral sclerosis	Song et al. (2023), Zou et al. (2023)
• The regulators of BECN1 core complex activity		
ATG14	Through BATS domain, a membrane curvature sensor can target the membrane structures of phagophores	Xiong et al. (2012), Yuan et al. (2024)
BCL2	Binds to BECN1 and blocks apoptosis	Kelekar and Thompson (1998)
RUBCN	A crucial component of autoimmunity and the immune response Regulates endosome maturation and acts as a negative regulator of autophagy and endocytic trafficking	Wong Y. et al. (2018), Yamamuro et al. (2022)
UVRAG	Its novel function in regulating autophagic and endosomal maturation is involved in the development of autophagosomes UVRAG operates by modulating autophagy through two sequential processes: BECN1-UVRAG is involved in the biogenesis of autophagosomes, whereas the class C/Vps complex is involved in the formation of autolysosomes	Liang et al. (2008), Shi et al. (2023)
• Phagophore expansion-associated proteins		
ATG12	E3-like complex that combines Atg8-family proteins to PE Ubiquitin-like protein; forms isopeptide bond with ATG5 during cell cycle progression and apoptosis	Rubinstein et al. (2011)
ATG16L1	An essential part of the E3-ligase-like phagophore expansion complex, which is necessary for autophagy’s catabolic process Interacts with the ATG12–ATG5 conjugate complex to generate a bilayer membrane that is necessary for phagophore expansion and autophagosome formation, hence playing a crucial part in the autophagy pathway	Salem et al. (2015), Ma et al. (2021), Taraborrelli et al. (2023)
ATG5	Performs roles in the development of autophagosomes and the regulation of autophagy. Acts as a mediator between serum-starvation-induced autophagy activation conditions and normal culture conditions	Li S. et al. (2021)
ATG7	E1-like enzyme; activation of Atg8-family proteins and ATG12 allowing conjugation to PE and ATG5, respectively	Collier et al. (2021)
• Atg8-family proteins		
GABARAP	Involved in the response to various cellular circumstances it is integrated into membranes, resulting in the development and maturation of autophagosomes. Also involved in cargo detection during selective autophagy	Park et al. (2019), Iriondo et al. (2023)
GABARAPL1	Involved in the movement of proteins or vesicles connected to several processes, including autophagy, tumor development, cell death, and proliferation Trafficking of receptors. Might support OPRK1 (opioid receptor kappa 1) and potentially other proteins by acting as a chaperone The LC3/GABARAP proteins have a variety of roles in autophagy, such as intracellular trafficking, phagocytosis, oncogenic/tumor suppressive actions, and cell motility. Enhance the susceptibility of cancer cells to ferroptosis, an iron-dependent form of programmed cell death; this prevents the development of metastasis by interfering with EMT	Grand et al. (2011), Beaumont et al. (2024)
GABARAPL2	Involved in reducing septic shock and inflammation brought on by CASP4/CASP11 (caspase 4; non-canonical inflammasome) that is dependent on GBP (guanylate binding protein). Acts at the end of autophagy by encouraging phagophore closure or autophagosome fusion with lysosomes	Mohan and Wollert (2018), Di et al. (2023)
• LC3 processing-related proteins		
ATG3	Improved E3 contact with the membrane, which permits liposome tethering in its early stages. A stress-induced autophagic enzyme that resembles an E2 that facilitates the conjugation of Atg8-family proteins to phosphatidylethanolamine (PE)	Popelka and Klionsky (2021), Iriondo et al. (2023)
ATG7	A target in the development of treatments that control autophagy, in cell biology and human illness, and in membrane trafficking processes that rely on LC3 lipidation. Inactivating ATG7 increases the efficacy of anti-cancer medicines in the treatment of lung and breast cancer cells by attenuating its capacity to sensitize tumor cells to cancer treatments	Collier et al. (2021)
ATG4A	Acts as an autophagy modulator	Nguyen et al. (2020), Li and Zan (2022)
ATG4B	Involved in autophagy signaling and associated with the advancement of several cancer types	Hu et al. (2023)
ATG4C	When additional ATG4 proteins are absent, in the priming and delipidation of Atg8-family proteins	Tamargo-Gómez et al. (2023)
• Autophagic selectivity		
BNIP3	BNIP3 (BCL2 interacting protein 3) overexpression in myeloma cells, with special reference to its impact on mitochondria and apoptosis. Suggested as a tumor marker. It is thought to be a viable target for stopping residual hepatocellular carcinoma from growing quickly and spreading after radiation therapy but is insufficient to ablate the cancer. Breast cancer growth metastasis linked to obesity is significantly reduced by BNIP3, thereby slowing the cancer’s progression	Macher-Goeppinger et al. (2017), Niu et al. (2019), Xu et al. (2019), Xiao et al. (2023)
BNIP3L	In the mitochondrial degradation pathway and PRKN (parkin RBR E3 ubiquitin protein ligase)-mediated mitophagy throughout the maturation phase of reticulocytes. Can inhibit MTORC1 to cause hypoxia colon cancer, brain ischemia, and autophagy	Gao et al. (2015), Han et al. (2020), Li et al. (2022)
NBR1	Involved in controlling the production of protein aggregates, the spread of malignant cells, and immune evasion	Marsh et al. (2020), Song et al. (2024)
PINK1	The PINK1 (PTEN induced kinase 1) signaling system regulates a number of essential processes in the biology of cancer cells, particularly those related to mitochondrial homeostasis and dynamics, such as fission and fusion, bioenergetics, and mitophagy, which, depending on the cellular context, can either promote or suppress tumor growth	Celis-Pinto et al. (2023)
PRKN	Involved in both mitophagy-dependent and -independent growth and metastasis of a number of cancer types	Zhang et al. (2024)
SQSTM1	A crucial molecule connected to several signaling pathways, oxidative reactions and inflammation that plays a role in autophagy. SQSTM1 accumulation is indicative of compromised autophagy, which is linked to the development of several malignancies, including HCC, and carcinogenesis	Abdel-Moety et al. (2022)

Linkage of the KEAP1 (kelch like ECH associated protein 1)-NFE2L2 system to autophagy is implied by the assembly of SQSTM1 on specific autophagic cargos, such as ubiquitinated organelles, and its subsequent phosphorylation in an MTORC1-dependent manner. The development of HCCs is facilitated by the continuous activation of NFE2L2 caused by the buildup of phosphorylated SQSTM1. Therefore, inhibitors of the connection between KEAP1 and phosphorylated SQSTM1 show potential as therapeutic drugs against human HCC. The KEAP1-NFE2L2 pathway and selective autophagy are interdependent (Ichimura et al., 2013; Inami et al., 2011).

## The role of autophagy in the development of tumors

Autophagy suppresses tumorigenesis by preserving physiological homeostasis and preventing cell conversion to malignancy in part by lowering the quantity of damaged mitochondria; the elimination of the latter helps prevent the formation of reactive oxygen species that can further cause damage of cellular components including DNA (Nazio et al., 2019; Jawad et al., 2023). Accordingly, inhibition of autophagy promotes oncogenesis and malignant transformation, and a high carcinogenic incidence (Takahashi et al., 2007; Jabir et al., 2015; Yun et al., 2020). BECN1 has been linked to the start of autophagy as well as a number of other cellular functions, including cell death, development, aging, and stress adaptation. Furthermore, BECN1 controls autophagic activity via interacting with other ATG proteins, the class III phosphatidylinositol 3-kinase (PtdIns3K) complex, and TP53/p53 (tumor protein p53), among other autophagy mediators, which in turn affects the initiation and progression of cancer (Yun et al., 2020). A reduction in BECN1 expression leads to both cancer growth and tumorigenesis (Vara-Perez et al., 2019), whereas BECN1 regulates autophagic activity to prevent the growth of tumors. BECN1 promotes autophagy and inhibits the growth of malignancies that are mediated by ERBB2/HER2 (erb-b2 receptor tyrosine kinase 2). Mitophagy is a crucial type of selective autophagy of mitochondria, removing damaged or abnormal organelles to maintain mitochondrial homeostasis (Chang et al., 2017). Defects in mitophagy can lead to mitochondrial damage, tumorigenesis, and tumor growth in different types of cancer (Vara-Perez et al., 2019). Mitophagy function varies depending on tumor stage, with mutations or functional changes causing an accumulation of impaired mitochondria and tumorigenesis (Yun et al., 2020). Mitophagy inhibits cancer during the early stages of carcinogenesis while maintaining the metabolic needs of healthy cells. Conversely, mitophagy promotes cell tolerance and accelerates tumor growth during the later stages of tumor development (Wang et al., 2020).

## Autophagy’s dual role in cancer

As discussed above, autophagy breaks down and recycles long-lived, misfolded or damaged proteins, and aberrant or damaged organelles to preserve cellular homeostasis (Huang T. et al., 2018; Onorati et al., 2018). Moreover, autophagy regulation protects against various types of cellular stressors, including starvation, hypoxia, DNA damage, and chemotherapeutic exposure, as well as fulfilling the metabolic needs of cells to maintain the functionality of organelles and cellular communication channels. As an integral process of cell physiology, both the maintenance of health and the development of diseases can be linked to autophagy or defects in the autophagy pathway. Numerous illnesses, such as cardio-related diseases (Zhang et al., 2023), neurological disease (Chu, 2019), gastrointestinal diseases (Zhang et al., 2023), lung diseases (Yun et al., 2020), cancer (Raudenska et al., 2021), and type II diabetes (Hashemi et al., 2023), are linked to abnormal autophagy. Both the initiation and spread of malignant tumors are influenced by tumor-suppressive autophagy. In contrast, the removal of aberrant cells and organelles, as well as the limitation of cell division and genetic instability in cancer, due to normal autophagy produce tumor inhibitory effects (Dong et al., 2019).

Numerous studies have suggested that autophagy has a dual role in the onset and progression of cancer (Li et al., 2020). However, there is an ongoing debate as to whether autophagy functions primarily as a pro- or anti-tumor mechanism (Zhang et al., 2023). Autophagy is involved in the quality control of proteins and organelles during the early stages of tumorigenesis (Barnard et al., 2016). It does this by preserving genomic stability, guarding against tissue damage over time, and preventing the accumulation of oncogenic proteins linked to inflammation. These actions prevent the initiation, proliferation, invasion, and metastasis of tumors (Guo et al., 2013). Research has shown that artificially limiting autophagy (for example, by *atg5* deletion in mice) enhances the early development of liver cancers, suggesting that tumor suppression is a key function of autophagy in hepatocytes (Takamura et al., 2011).

**FIGURE 2:**
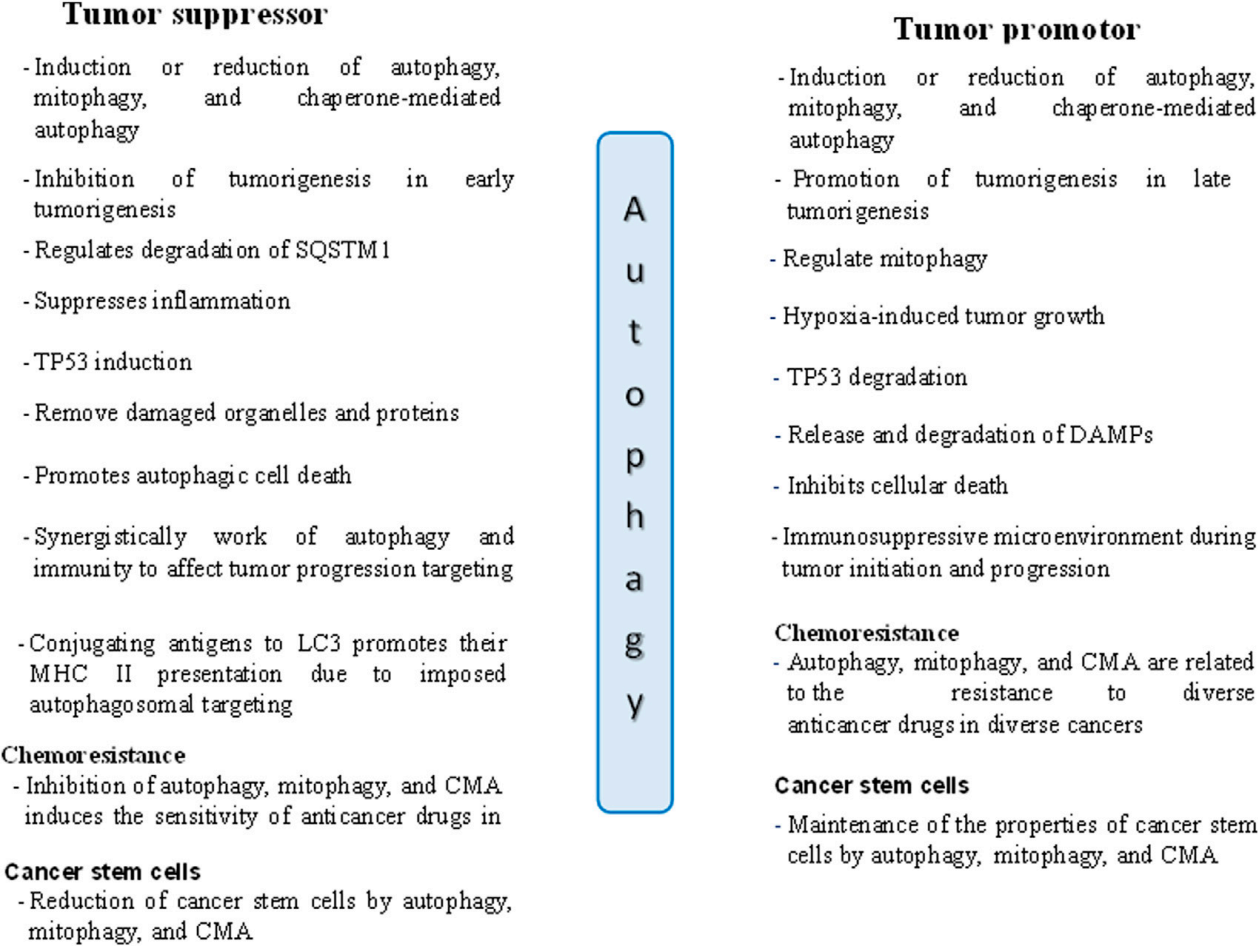
The dual roles of autophagy in tumor suppression and promotion in cancer cells. DAMPs: Danger/damage associated molecular patterns.

In contrast, when a tumor reaches an advanced stage, autophagy becomes a shield for the tumor cells, protecting against DNA damage and increasing the survival of cancer cells by causing resistance to drugs (Wu et al., 2012; Zhang et al., 2023). Thus, autophagy promotes cancer cells by triggering chemoresistance and satisfying the growing metabolic requirements of cancer cells (Anderson and Macleod, 2019; Xiong et al., 2019). According to Liu et al. (2018), autophagy increases the expression of the transcription factor NANOG (Nanog homeobox) and suppresses TP53, encouraging hepatocarcinogenesis in benign liver tumors in a process involving hepatoma stem cells (Liu et al., 2018). The dual roles of autophagy in cancer, both supporting and suppressing tumor growth, are illustrated in Figure 2 (Yun et al., 2020).

## Roles of autophagy in HCC

**FIGURE 3:**
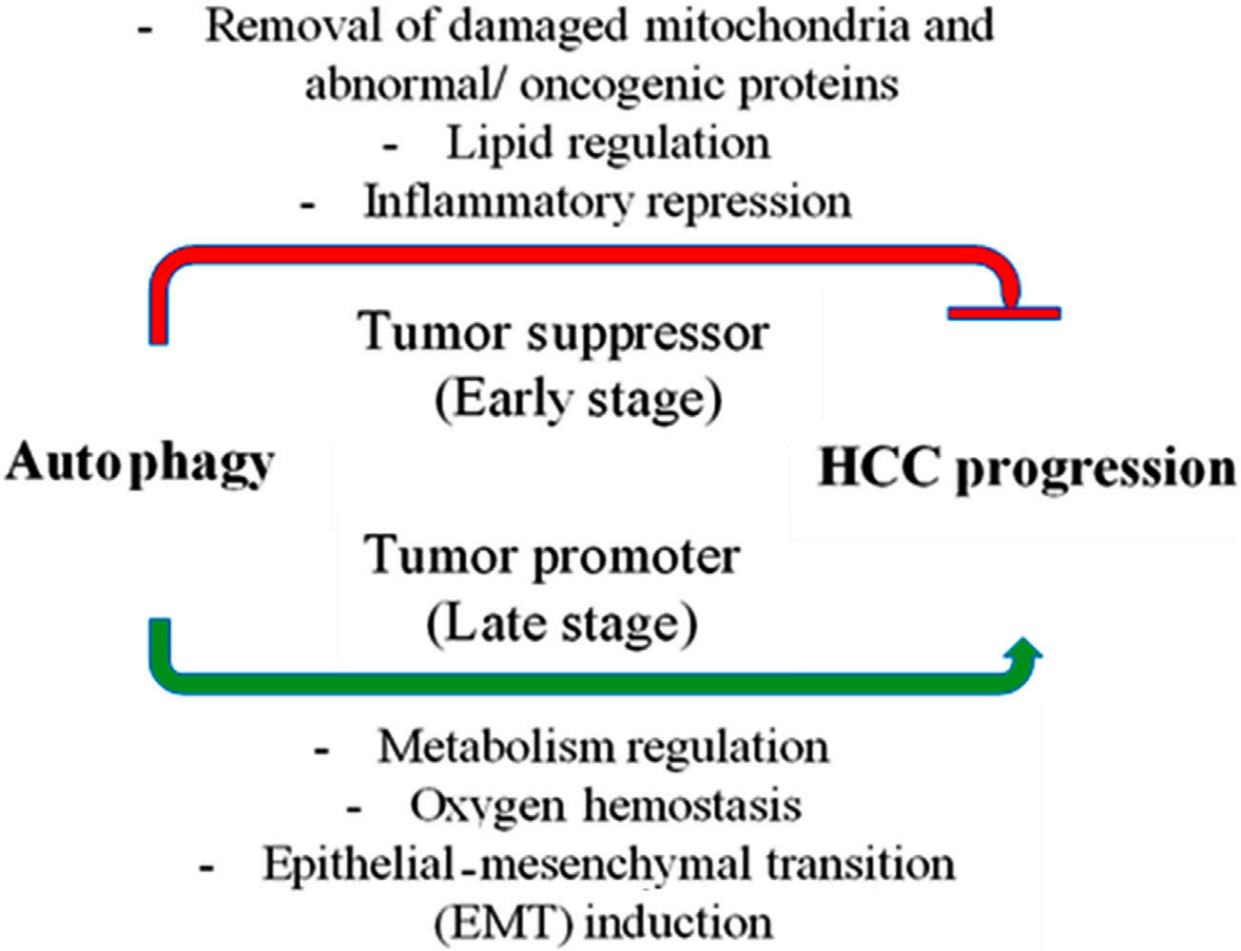
Autophagy’s role in hepatocellular carcinoma (HCC). In the early stages of cancer, autophagy suppresses tumors by removing damaged mitochondria and abnormal proteins, controlling hepatic lipid metabolism, and reducing inflammation (represented by the red line). However, once cancer has taken hold, autophagy becomes a tumor promoter by controlling metabolism and preserving the oxygen equilibrium, which helps cancer cells survive. Additionally, autophagy promotes the progression of the disease by inducing the epithelial-mesenchymal transition (EMT).

In HCC, autophagy has a conflicting function, both in preventing early-stage carcinogenesis and in accelerating the growth of tumors in later stages (Ni et al., 2014). This dual role demonstrates how difficult it is to target autophagy in the treatment of HCC. Autophagy and the control of the development and progression of HCC are mediated by autophagy-associated genes, non-coding RNAs, and associated signaling pathways (Figure 3) (Yang et al., 2019). Autophagy has two roles in the onset and progression of hepatocellular cancer and many factors trigger the activation of hepatic autophagy. First, autophagy can function as a tumor suppressor during the initiation stage of hepatoneogenesis by reducing inflammation, SQSTM1 accumulation, the oxidative stress response, and ultimately genomic instability and through autophagic cell death. Second, however, at other stages of hepatoneogenesis, autophagy can promote cancer through its cytoprotective functions (Lee and Jang, 2015). Autophagy’s precise role in HCC is debatable and remains incompletely understood. Extensive investigation is necessary to comprehend the function of autophagy in the progression of HCC.

## Autophagy and proteins associated with autophagy in HCC

**FIGURE 4:**
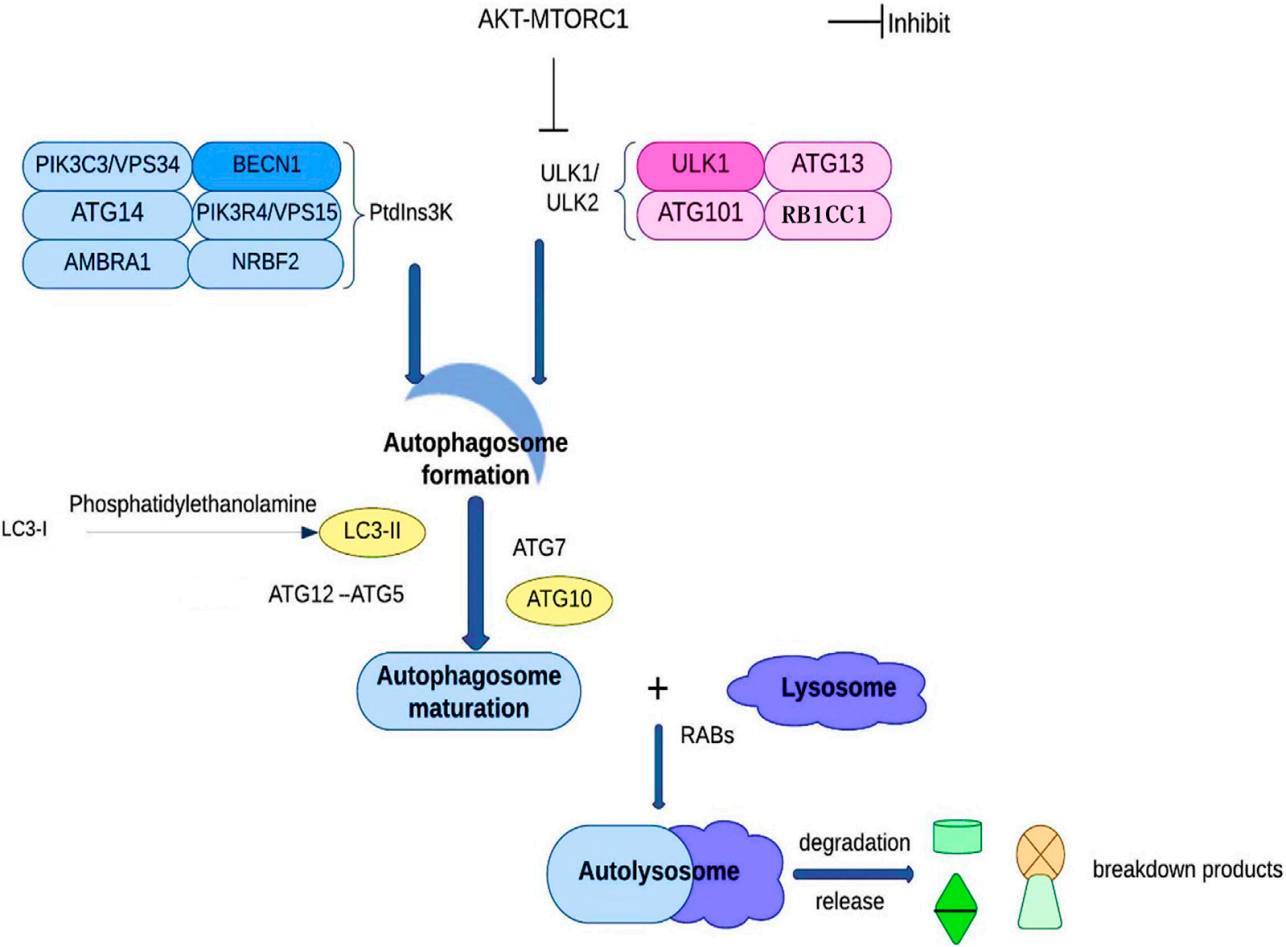
Autophagy and autophagic proteins. See the text for details.

In order to maintain the equilibrium of cell component production and breakdown, autophagy involves the following steps: phagophore formation, autophagosome maturation, autolysosome formation, and cargo degradation (Levy et al., 2017). Autophagy contributes to the preservation of the nitrogen balance and the equilibrium of the cell environment by using the lysosomal route to break down macromolecules when the cell is malnourished (Kim and Lee, 2014; Wu et al., 2021). Based on the transport pathways and on their capacity to break down specific cargo, they can be categorized as reticulophagy (endoplasmic reticulum), pexophagy (peroxisomes), ribophagy (ribosomes), xenophagy (invasive microbes), mitophagy (mitochondria), etc. (Chu, 2019; Kirkin, 2020). Cargo sequestration, autophagosome development and autolysosome-dependent degradation are the three main components of autophagy. ATG101, ATG13, RB1CC1, and ULK1/ULK2 make up the ULK1/ULK2 complex during the early stages of autophagy development. The PtdIns3K complex, which has the lipid kinase PIK3C3/VPS34 at its core, plays a critical role by synthesizing phosphatidylinositol-3-phosphate on the phagophore, allowing the recruitment of other proteins leading to the autophagosome’s development. Subsequently, LC3-I is converted into LC3-II through conjugation to phosphatidylethanolamine, along with ATG12–ATG5 conjugation, and the action of specific receptors such as SQSTM1 to promote autophagosome maturation. Ultimately, the autophagosome fuses with a lysosome under the direction of RAB proteins to carry out the cargo breakdown and release (Figure 4) (Wu et al., 2021).

## Autophagy and HCC drug resistance

Chemoresistance in HCC has proven to be a difficult problem in recent years, but it can be overcome by stimulating oxidative stress and inhibiting mitochondrial respiration. In addition, the interplay between different factors can influence autophagy levels, affecting cancer development and progression. For example, HCC is associated with ICMT (isoprenylcysteine carboxyl methyltransferase), NFE2L2, and USP7 (ubiquitin specific peptidase 7) via a number of pathways, including control over cell survival, proliferation, and metabolism. In HCC, overexpression of ICMT results in doxorubicin resistance and inhibits apoptosis (Kiruthiga et al., 2020). Conversely, cancer cells become more drug sensitive when NFE2L2/Nrf2 is overexpressed because it causes chemoresistance in HCC (Cai et al., 2020), whereas medication sensitivity is caused by downregulation of USP7, which also inhibits HCC cell proliferation and metastasis (Zhang et al., 2020). However, depending on the particular signaling pathways involved and the setting, the precise interaction among these components in HCC may differ. Thus, to fully understand the connections between ICMT, NFE2L2, and USP7 in HCC, more research is required (Ying et al., 2023). One major pathogenic role for NFE2L2 deregulation in HCC is hypothesized. When defective autophagy occurs under specific pathophysiological conditions, such as oxidative stress, NFE2L2 activation follows, which has negative effects that promote HCC survival and proliferation. Through autophagic pathways, NFE2L2 is involved in the migration, invasion, and proliferation of HCC. For example, NFE2L2 is negatively controlled by KEAP1 (kelch like ECH associated protein 1), which contributes to HCC carcinogenesis by increasing the production of ROS; autophagy may help HCC cells undergo an oxidative metabolic reprogramming (Moon et al., 2012).

Comprehensive evidence substantiates the association between autophagy and drug resistance, development, migration, and cancer (Russo and Russo, 2018). However, it is still unknown how, particularly in cases of treatment resistance, autophagy flux and tumor cell state control autophagy to either become a tumor guardian or a tumor killer (Sun et al., 2013). Medication therapy stimulates autophagy and increases its flux; nevertheless, elevated autophagy flux also stimulates drug resistance or, by modifications to autophagy-related proteins, causes tumor cell death (Sheng et al., 2018).

Because autophagy helps tumor cells survive under therapeutic stress, it is also thought to be a significant factor of drug resistance (Lei et al., 2024). Therefore, HCC cells may become more sensitive to chemotherapeutic treatments if autophagy is suppressed (Wang et al., 2017).

To put it briefly, learning more about how autophagy influences drug resistance in HCC, such as the initially prescribed drug, traditional chemotherapeutic medications, and cutting-edge anticancer agents, is extremely important and needs further research.

## Conclusion

The main treatment for HCC, a common malignant tumor and a major subtype of liver cancer, is surgical excision. 65%–70% of patients are at a middle or advanced stage and need chemotherapy. Treatment failure, however, might result from drug resistance to chemotherapeutic agents. Drug efflux pump transport, DNA repair ability, hereditary variables, and adaptive responses are some of the mechanisms underlying drug resistance. In both healthy and malignant cells, autophagy is an essential biochemical process that has many applications based on the situation. While autophagy helps normal cells break down toxic substances and ageing organelles, cancer cells can either stimulate or block autophagy in order to increase their chances of surviving. Drug resistance is a complex process involving autophagy, with dysregulation of autophagy activation being one of the contributing factors. Dysregulation of the expression of BECN1 or LC3, or SQSTM1-induced activation of autophagy contribute to medication resistance. Targeting proteins related to autophagy, signaling pathways, and exosomes can help reverse drug resistance. Autophagy affects both survival and death in HCC; pro-survival autophagy increases cell viability, while pro-death autophagy hinders tumor growth. Increasing sensitivity to medications and radiation can be achieved by targeting autophagy, which may benefit HCC patients’ prognosis, survival, and course of treatment. Treating advanced and metastatic stages of HCC presents obstacles, including early diagnosis and therapeutic resistance.

However, the intricacy of autophagy, the heterogeneity of the disease, and the need for customized treatment make it difficult to combine autophagy inhibition with currently available HCC medications. There are targeted therapies that rely on the role of autophagy in HCC resistance using drugs such as sorafenib, cisplatin, 5-fluorouracil, oxaliplatin, and Pirarubicin (THP-adriamycin) and doxorubicin. To improve the sensitivity of HCC to anticancer medications, a better knowledge of autophagy’s role in drug resistance is necessary. Additionally, further multicenter medical studies are needed for the therapy of anti-HCC in conjunction with the suppression of autophagy.
